# Disrupting AMPK-Glycogen Binding in Mice Increases Carbohydrate Utilization and Reduces Exercise Capacity

**DOI:** 10.3389/fphys.2022.859246

**Published:** 2022-03-22

**Authors:** Natalie R. Janzen, Jamie Whitfield, Lisa Murray-Segal, Bruce E. Kemp, John A. Hawley, Nolan J. Hoffman

**Affiliations:** ^1^ Exercise and Nutrition Research Program, Mary MacKillop Institute for Health Research, Australian Catholic University, Melbourne, VIC, Australia; ^2^ St Vincent’s Institute of Medical Research and Department of Medicine, University of Melbourne, Fitzroy, VIC, Australia

**Keywords:** AMP-activated protein kinase, carbohydrate binding module, glycogen, skeletal muscle, exercise, energy utilization, metabolism

## Abstract

The AMP-activated protein kinase (AMPK) is a central regulator of cellular energy balance and metabolism and binds glycogen, the primary storage form of glucose in liver and skeletal muscle. The effects of disrupting whole-body AMPK-glycogen interactions on exercise capacity and substrate utilization during exercise *in vivo* remain unknown. We used male whole-body AMPK double knock-in (DKI) mice with chronic disruption of AMPK-glycogen binding to determine the effects of DKI mutation on exercise capacity, patterns of whole-body substrate utilization, and tissue metabolism during exercise. Maximal treadmill running speed and whole-body energy utilization during submaximal running were determined in wild type (WT) and DKI mice. Liver and skeletal muscle glycogen and skeletal muscle AMPK α and β2 subunit content and signaling were assessed in rested and maximally exercised WT and DKI mice. Despite a reduced maximal running speed and exercise time, DKI mice utilized similar absolute amounts of liver and skeletal muscle glycogen compared to WT. DKI skeletal muscle displayed reduced AMPK α and β2 content versus WT, but intact relative AMPK phosphorylation and downstream signaling at rest and following exercise. During submaximal running, DKI mice displayed an increased respiratory exchange ratio, indicative of greater reliance on carbohydrate-based fuels. In summary, whole-body disruption of AMPK-glycogen interactions reduces maximal running capacity and skeletal muscle AMPK α and β2 content and is associated with increased skeletal muscle glycogen utilization. These findings highlight potential unappreciated roles for AMPK in regulating tissue glycogen dynamics and expand AMPK’s known roles in exercise and metabolism.

## Introduction

The AMP-activated protein kinase (AMPK) is an important regulator of energy utilization and metabolic homeostasis. AMPK regulates multiple metabolic pathways that help maintain and restore energy balance during energy stress, including glycolysis as well as fatty acid synthesis and oxidation. The AMPK heterotrimeric complex consists of a catalytic α subunit and β and γ regulatory subunits. The β subunit scaffolds the heterotrimer and contains a carbohydrate binding module (CBM) which allows AMPK to bind glycogen (Hudson et al., 2003; Polekhina et al., 2003; Polekhina et al., 2005; McBride et al., 2009). In rodents, the β1 isoform is predominantly expressed in liver while the β2 isoform, which has the capacity to bind glycogen more tightly (Koay et al., 2010; Mobbs et al., 2017), is predominantly expressed in skeletal muscle (Janzen et al., 2018).

Glycogen serves as a critical energy substrate and is predominantly stored in liver and skeletal muscle. In response to energy stress such as exercise, glycogen is mobilized into glycolytic intermediates and resynthesized to help restore liver and/or skeletal muscle energy levels. In rodents, liver glycogen stores are used to maintain euglycemia in both the post-prandial and energy-depleted states, while skeletal muscle stores are preserved for strenuous exercise (Baldwin et al., 1973; Reitman et al., 1973; Terjung et al., 1973). The contribution of glycogen to total energy expenditure becomes higher with increasing exercise intensity (van Loon et al., 2001), and depletion of skeletal muscle glycogen ultimately results in fatigue (Philp et al., 2012; Nielsen and Ortenblad 2013; Ortenblad et al., 2013).

Glycogen interactions mediated by the AMPK β subunit CBM have been hypothesized to serve an energy-sensing role, whereby AMPK senses stored glycogen levels and regulates metabolic pathways to maintain cellular energy homeostasis (McBride et al., 2009; McBride and Hardie 2009), such as during exercise. In support of this theory, whole-body AMPK β2 knockout (KO) mice have reduced maximal treadmill running speed, and while WT mice had significant reductions in skeletal muscle glycogen post-exercise, AMPK β2 KO mice had no change in skeletal muscle glycogen content following a maximal test (Dasgupta et al., 2012). Additionally, skeletal muscle specific AMPK β1/β2 KO mice have reduced maximal running speed, concomitant with increased reliance on fat as an energy substrate (O'Neill et al., 2011). AMPK β2 knock-in (W98A KI) mice with disrupted β2 subunit isoform glycogen binding have impaired maximal running speed, although this effect was not observed in β1 (W100A KI) mice (Hoffman et al., 2020). Single AMPK KI mutation utilized to disrupt β1 or β2 AMPK-glycogen binding led to reduced tissue AMPK levels in liver or skeletal muscle, respectively, suggesting that glycogen binding may play a role in maintaining tissue AMPK content (Hoffman et al., 2020). AMPK double knock-in (DKI) mice with chronic whole-body disruption of glycogen binding capacity via simultaneous mutation of both β1 and β2 isoforms display increased adiposity, increased rates of carbohydrate (CHO) oxidation and altered tissue glycogen dynamics in the fed and resting state and in response to fasting, concomitant with reductions in AMPK content in liver and skeletal muscle (Janzen et al., 2021).

Whether reduced tissue AMPK in DKI mice and the accompanying alterations in whole-body substrate utilization patterns and tissue metabolism affect energy utilization during exercise and ultimately exercise capacity in these mice remains unknown. Accordingly, we utilized this DKI mouse model with whole-body disruption of AMPK-glycogen binding to determine the effects of the DKI mutation on maximal running speed and fuel utilization during exercise. We hypothesized that DKI mice would have reduced maximal running speed and increased CHO oxidation rates during treadmill exercise compared to WT.

## Results

### Double Knock-In Mice Have Reduced Maximal Running Speed

**FIGURE 1:**
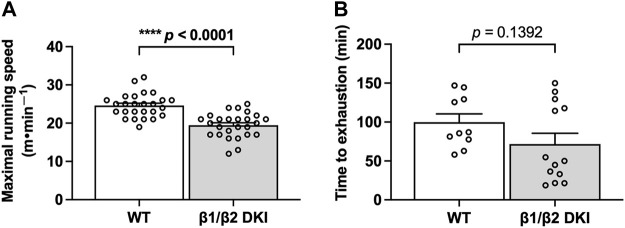
The double knock-in (DKI) mutation used to disrupt whole-body AMPK-glycogen binding in mice reduces maximal running speed but does not alter time to exhaustion at submaximal running speed. **(A)** Maximal running speed at a 0° treadmill incline in wild type (WT) and DKI mice (*n* = 26); **(B)** Time to exhaustion at 70% of individual maximal running speed at a 0° incline (*n* = 10–13). Male mice, 17–21 wk ****p < 0.0001.

To determine whether the DKI mutation utilized to disrupt whole-body AMPK-glycogen binding alters exercise capacity, DKI and wild type (WT) mice were subjected to treadmill running tests at a 0° incline to determine maximal running speed and time to exhaustion at 70% of individual maximal running speed. DKI mice had significantly lower maximal running speed compared to WT (Figure 1A), while the time to exhaustion was similar between genotypes (Figure 1B).

### Double Knock-In Mice Display Increased Carbohydrate Utilization During Submaximal Exercise

**FIGURE 2:**
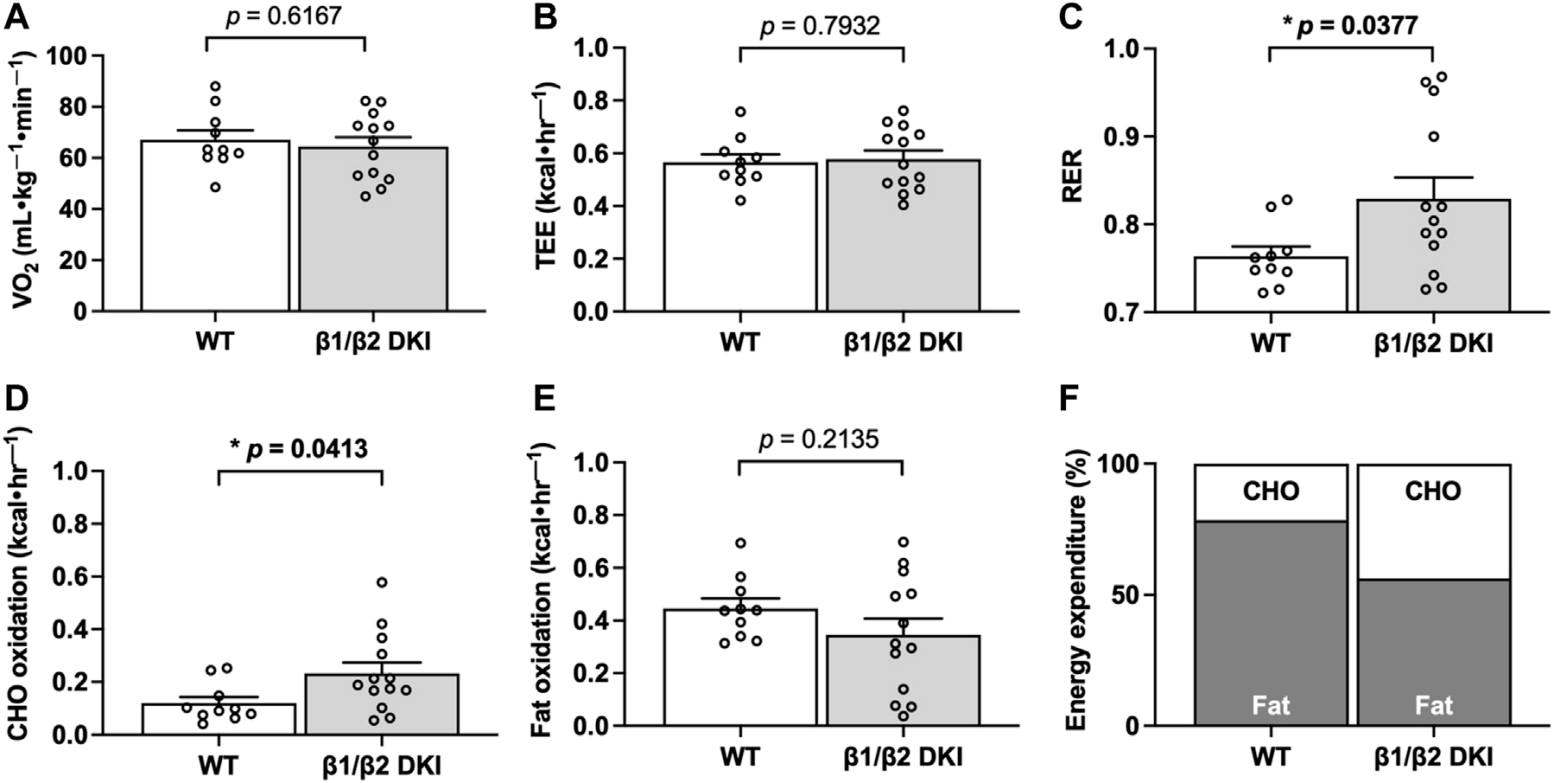
DKI mice have increased respiratory exchange ratio (RER) and carbohydrate (CHO) oxidation rates during exercise calorimetry experiments. Respiratory gases (VO_2_ and VCO_2_) were measured every min and used to calculate RER, total energy expenditure (TEE), and substrate utilization during exercise calorimetry treadmill running at 60% of individual maximal running speed. Data presented are the average over the final 5 min of exercise. **(A)** VO_2_; **(B)** TEE; **(C)** RER; **(D)** CHO oxidation rates; **(E)** Fat oxidation rates; **(F)** Percent contribution of fat and CHO oxidation rates to TEE. Male mice, 17–20 weeks, *n* = 11–13. *p < 0.05.

To determine if differences in patterns of substrate utilization contribute to the observed reduction in maximal exercise capacity in DKI versus WT mice, a subset of animals underwent exercise calorimetry testing using a treadmill equipped with indirect calorimetry. Continuous running at 60% of maximal running speed was used to ensure mice could complete the running protocol and to promote a mixed utilization of CHO- and fat-based substrates (Petrosino et al., 2016; Ishihara and Taniguchi 2018). While there were no observed differences in relative oxygen consumption (VO_2_; Figure 2A) or total energy expenditure (TEE; Figure 2B) during submaximal running between genotypes, DKI mice displayed an increase in respiratory exchange ratio (RER; Figure 2C), indicating increased reliance on CHO fuels during running compared to WT. In line with the observed increase in RER, DKI mice displayed a two-fold increase in calculated rates of CHO oxidation during submaximal running compared to WT mice (Figure 2D), while there were no differences between genotypes in calculated rates of fat oxidation (Figure 2E). This resulted in CHO oxidation accounting for nearly 45% of TEE in DKI mice–double the percentage observed in WT (Figure 2F). Meanwhile, fat oxidation accounted for 56% of TEE in DKI mice, compared to 78% of TEE in WT mice (Figure 2F).

### Double Knock-In Mice Have Similar Changes in Circulating Substrates in Response to a Maximal Running Test

**FIGURE 3:**
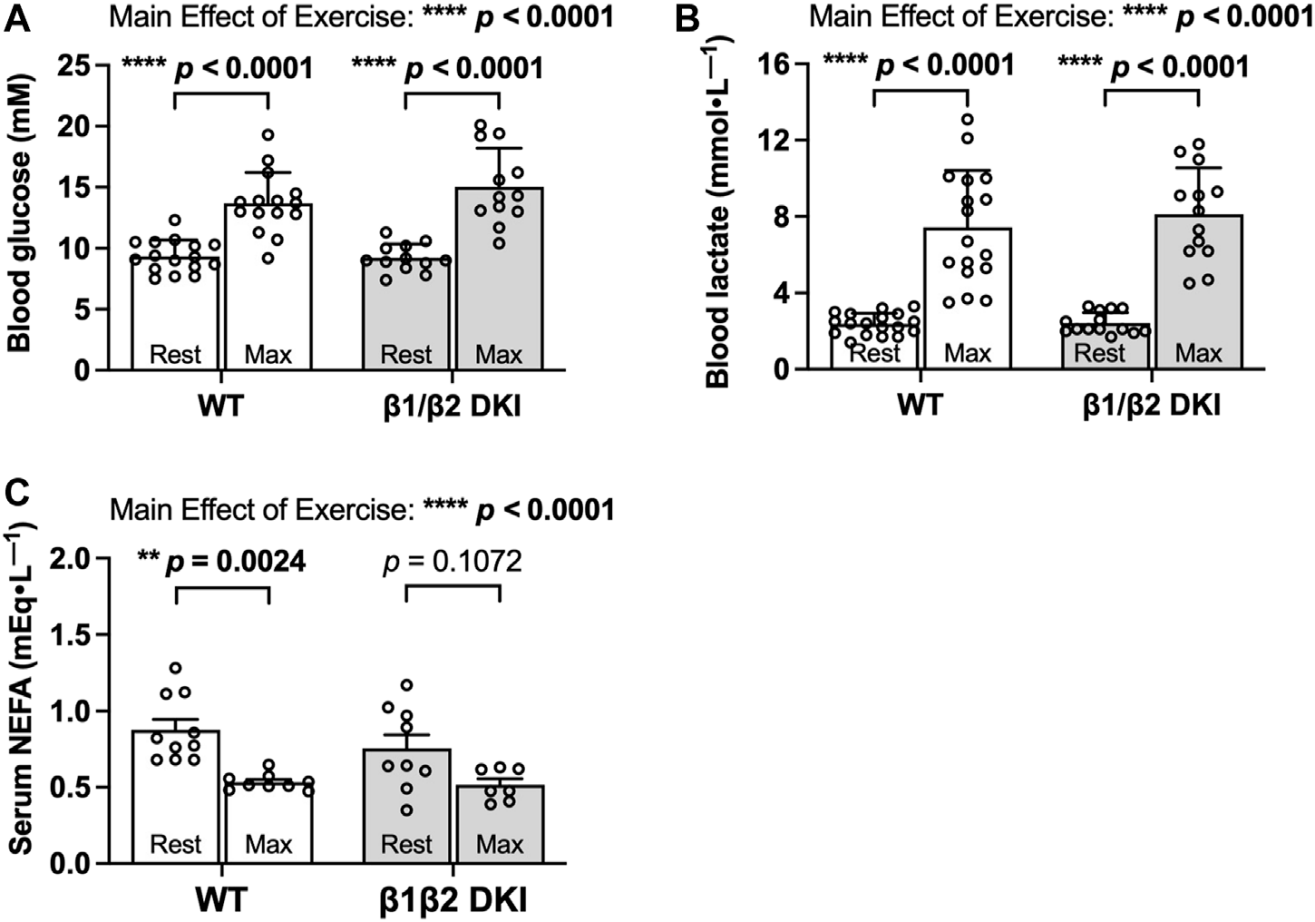
DKI and WT mice have similar circulating glucose, lactate, and non-esterified fatty acid (NEFA) levels following a maximal running test at a 5° incline. **(A)** Blood glucose and **(B)** lactate were measured via tail tip bleeding the morning before and immediately following the maximal exercise test at a 5° incline. Fed male mice, 17–20 weeks, *n* = 12–18. **(C)** Blood was collected via retro-orbital bleed from mice immediately before and after completing the maximal running test, and serum NEFA levels were assessed. Male mice, 17–20 weeks, n = 7–10. **p < 0.01, ****p < 0.0001.

To determine if differences in circulating substrates contributed to the observed changes in maximal running speed and substrate oxidation rates during exercise calorimetry experiments in DKI mice, blood glucose, lactate and serum non-esterified fatty acid (NEFA) levels were assessed. Fed, resting blood glucose levels and increases in blood glucose concentration following a maximal running test at a 5° incline were similar between WT and DKI (Figure 3A). WT and DKI mice also had similar resting lactate levels and similar increases in lactate concentration following the maximal running test at a 5° incline (Figure 3B) despite differences in absolute maximal running speed at a 0° incline. While circulating NEFA levels were similar between WT and DKI mice in the resting state, only WT mice displayed a significant decrease in NEFA concentrations in response to maximal running at a 5° incline (Figure 3C). However, NEFA levels following a maximal running test were similar between WT and DKI mice (Figure 3C). Together, these results suggest that alterations in whole-body substrate availability are not necessarily driving the observed changes in patterns of fuel utilization during exercise calorimetry.

### Double Knock-In Mice Have Similar Changes in Tissue Glycogen Levels Compared to Wild Type in Response to a Maximal Running Test Despite Less Total Running Time

**FIGURE 4:**
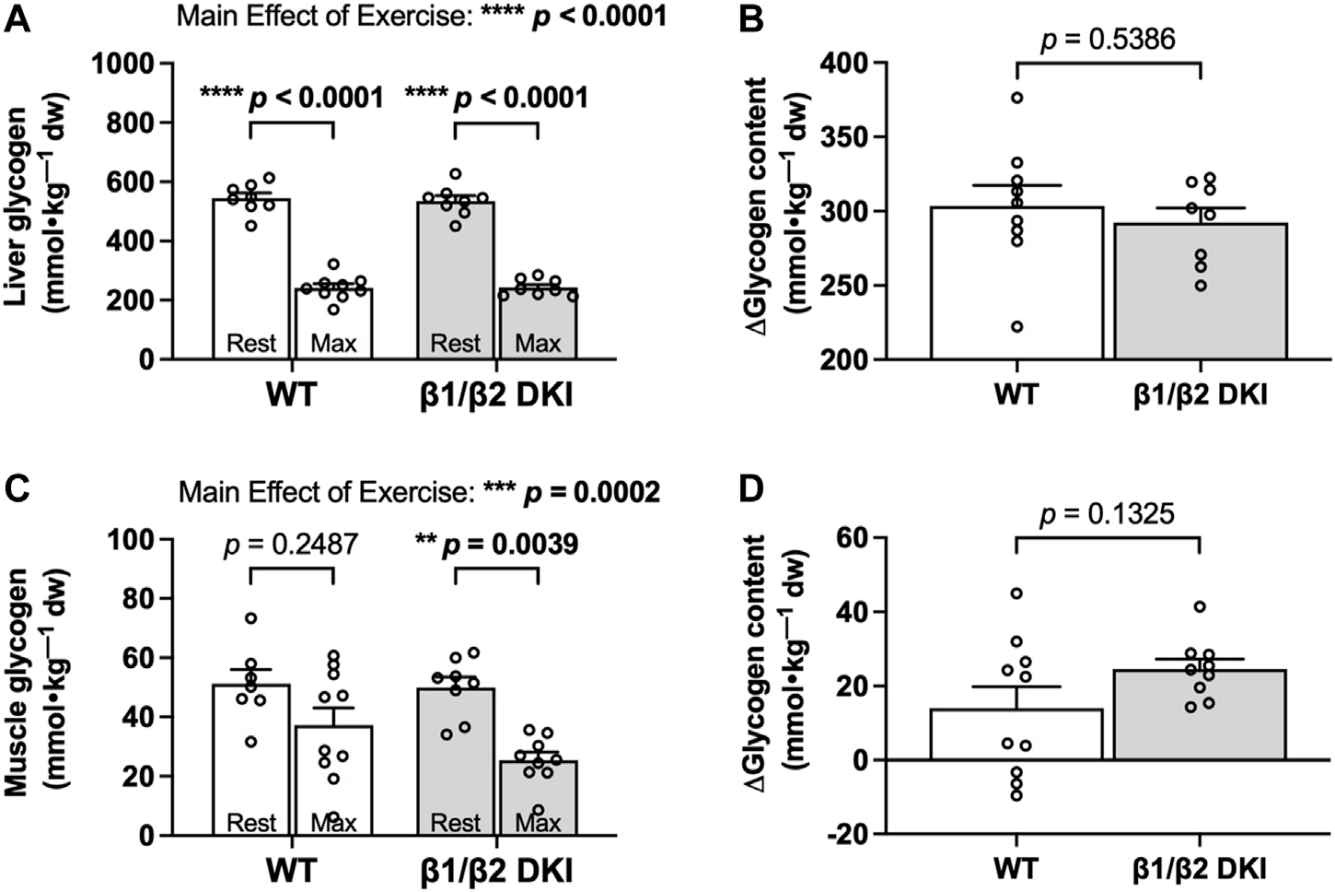
DKI and WT mice display similar changes in liver and skeletal muscle glycogen following a maximal running test at a 5° incline. Glycogen content was assessed in **(A)** liver and **(C)** skeletal muscle from rested mice and maximally exercised mice. Change in glycogen (Δglycogen content) in **(B)** liver and **(D)** skeletal muscle was determined by the difference between individual glycogen content of maximally exercised mice and the average glycogen content of rested mice from the same genotype. Male mice, 17–20 weeks, *n* = 7–10. **p < 0.01, ***p < 0.001, ****p < 0.0001.

Given the increased rates of CHO oxidation observed in DKI mice during exercise calorimetry experiments, we next determined if this increase was associated with a greater utilization of glycogen during exercise in the primary glycogen-storing tissues liver and skeletal muscle. Liver glycogen concentration in the resting state was similar between WT and DKI mice and declined to similar levels following a maximal running test at a 5° incline in both genotypes (Figure 4A). Change in glycogen (Δ glycogen content) in liver and skeletal muscle was then determined as the difference between individual glycogen content of maximally exercised mice and the average glycogen content of rested mice from the same genotype. WT and DKI mice had a similar absolute change in liver glycogen content from rest to following a maximal running test (Figure 4B). In skeletal muscle, resting glycogen concentration was similar between genotypes, but DKI mice displayed a significant reduction in skeletal muscle glycogen following a maximal running test versus rested DKI mice (Figure 4C). However, there were no differences detected in skeletal muscle glycogen concentration between WT and DKI mice following a maximal running test (Figure 4C). This similar level of depletion corresponded to a similar absolute change in glycogen content in skeletal muscle (Figure 4D). However, despite a ∼21% reduction in maximal running speed–and therefore, a 10 min reduction in overall treadmill running time–DKI mice had similar levels of muscle glycogen depletion compared to WT following a running test to exhaustion.

**FIGURE 5:**
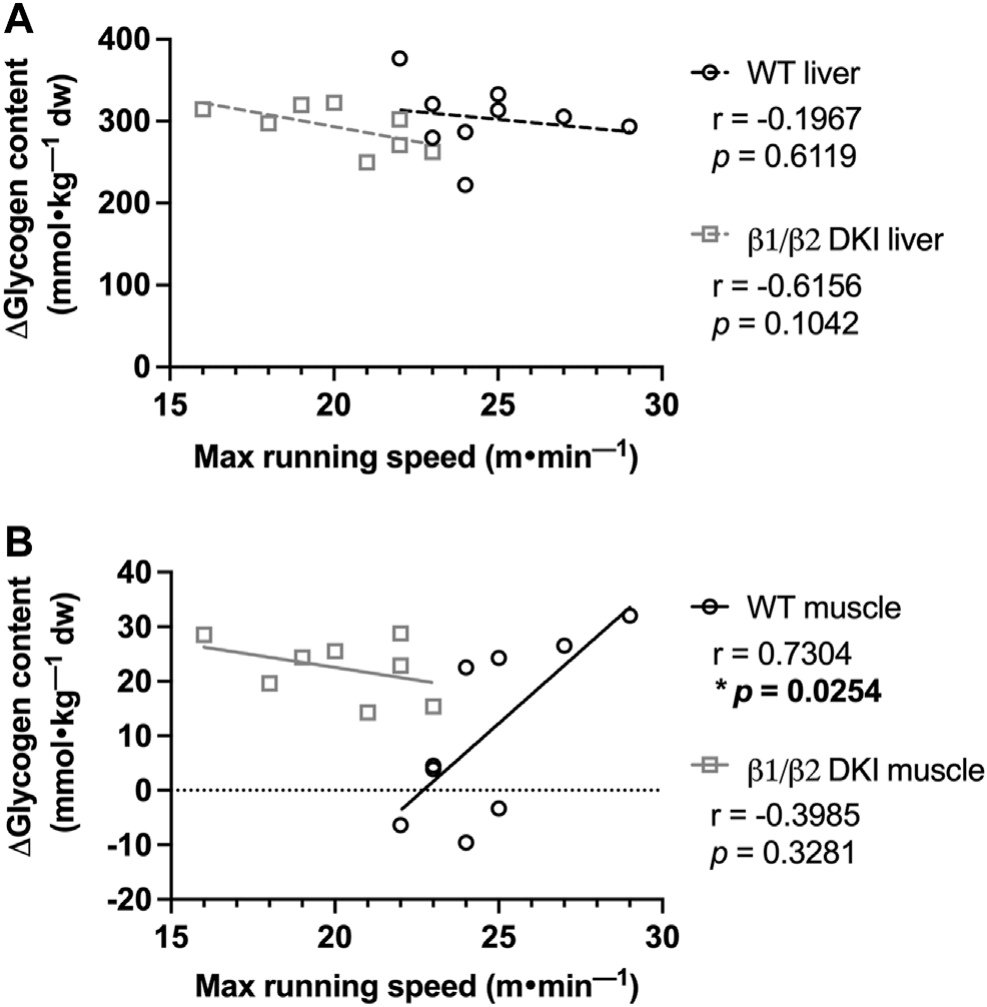
Maximal running speed at a 5° incline is positively correlated with skeletal muscle glycogen utilization following maximal running in WT but not DKI mice. **(A)** Correlation between maximal running speed and change in liver glycogen was not significant in either WT or DKI mice. **(B)** WT mice display a significant positive correlation between maximal running speed and change in skeletal muscle glycogen between the rested state and following maximal treadmill running, which was not observed in DKI mice. Male mice, 17–20 weeks, *n* = 8–9. *p < 0.05.

We next examined the potential relationship between levels of glycogen depletion and onset of fatigue during a maximal running test at a 5° incline. The change between mean tissue glycogen content of resting mice and individual tissue glycogen content of exercised mice following a maximal running test (i.e., Δ glycogen content) was determined and compared with the individual maximal running speed for each mouse. There was no significant correlation observed between change in liver glycogen and maximal running speed for either WT or DKI mice (Figure 5A).

In contrast, there was a significant positive correlation between change in skeletal muscle glycogen and maximal running speed at a 5° incline in WT mice (Figure 5B), indicating that WT mice which utilized more skeletal muscle glycogen reached higher maximal running speeds. However, there was no correlation between change in skeletal muscle glycogen and maximal running speed in DKI mice (Figure 5B), suggesting that higher skeletal muscle glycogen utilization did not lead to higher maximal running speed in DKI mice.

### Altered Patterns of Substrate Utilization in Double Knock-In Mice are Not Due to Changes in GLUT4, CPT1b or Mitochondrial Protein Content in Skeletal Muscle

**FIGURE 6:**
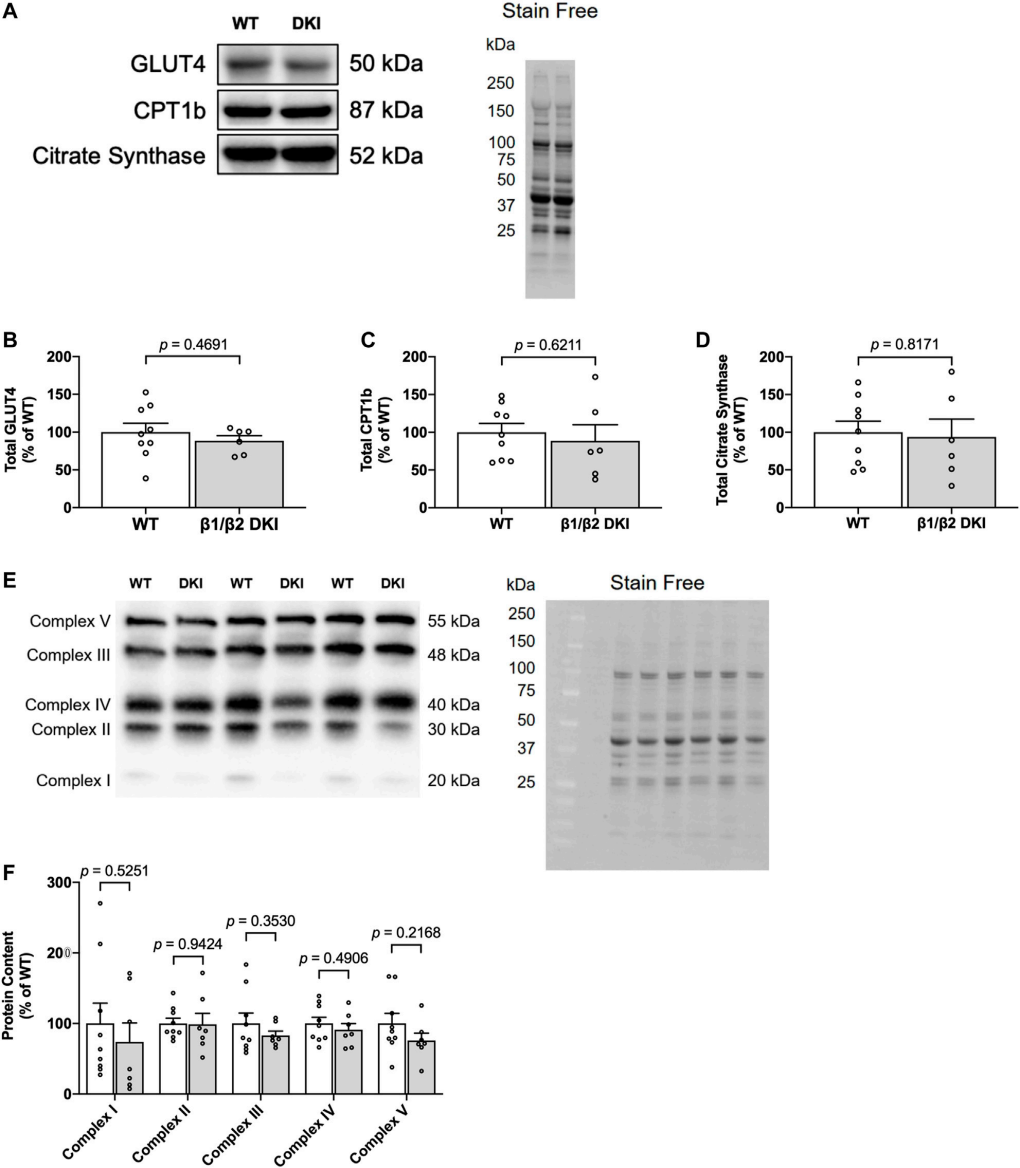
DKI mouse skeletal muscle displays similar content of proteins associated with glucose uptake and protein markers of mitochondrial content relative to WT. **(A)** Representative immunoblots and stain free image of GLUT4, CPT1b, and citrate synthase in WT and DKI skeletal muscle; **(B)** Quantified total GLUT4; **(C)** Quantified total CPT1b; **(D)** Quantified total citrate synthase; **(E)** Representative immunoblots and stain free image of mitochondrial oxidative phosphorylation (OXPHOS) complex proteins in WT and DKI skeletal muscle; **(F)** Quantified OXPHOS complex protein content. Male mice, 20–32 weeks, *n* = 7–9.

To further investigate the observed genotype differences in patterns of substrate utilization during exercise calorimetry experiments, and the lack of correlation between change in skeletal muscle glycogen content and maximal running speed at a 5° incline in DKI mice, markers of glucose transport (glucose transporter type 4 [GLUT4]), fat transport (carnitine palmitoyltransferase Ib [CPT1b]) and mitochondrial content (citrate synthase and oxidative phosphorylation [OXPHOS] complex proteins) were assessed in skeletal muscle from resting WT and DKI mice (Figures 6A,E). Skeletal muscle protein content of GLUT4 (Figure 6B), CPT1b (Figure 6C), citrate synthase (Figure 6D) and mitochondrial OXPHOS complexes (Complexes I, II, III, IV and V; Figure 6F) were similar between genotypes.

### Double Knock-In Mutation Reduces Skeletal Muscle AMP-Activated Protein Kinase α and β2 Content but Does Not Impair Relative AMP-Activated Protein Kinase Phosphorylation and Downstream Signaling in Response to a Maximal Running Test

**FIGURE 7:**
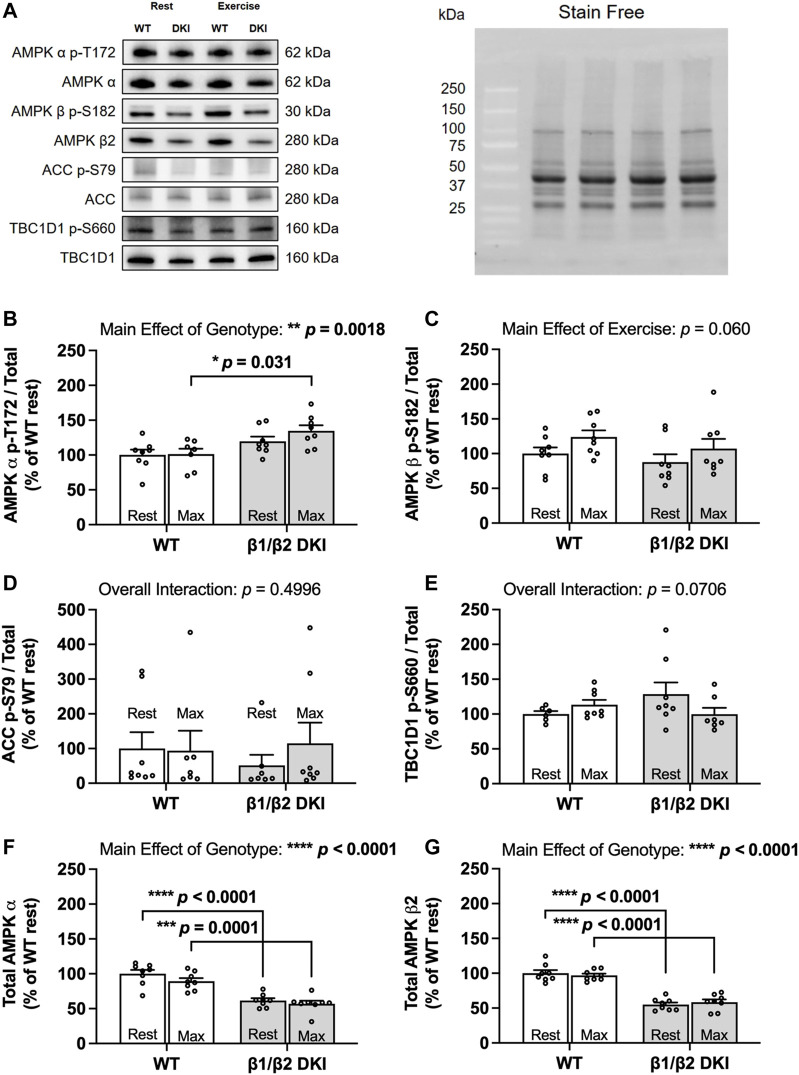
DKI mice have reduced skeletal muscle AMPK α and β2 content but intact AMPK, ACC and TBC1D1 signaling in response to a maximal running test at a 5° incline versus WT. Skeletal muscles were collected from rested and maximally exercised male WT and DKI mice, and phosphorylation of AMPK and downstream substrates was assessed using Western blotting. **(A)** Representative immunoblots of p-T172 and total AMPK, p-S182 and total AMPK β, p-S79 and total acetyl-CoA carboxylase (ACC) and p-S660 and total TBC1 domain family member 1 (TBC1D1) with representative stain free image. Quantified relative **(B)** AMPK p-T172, **(C)** AMPK β p-S182, **(D)** ACC p-S79, and **(E)** TBC1D1 p-S660; **(F)** Total AMPK α; **(G)** Total AMPK β. Male mice, 17–20 weeks, *n* = 6–8. *p < 0.05, **p < 0.01, ***p < 0.001, ****p < 0.0001.

Given AMPK’s role as a cellular energy regulator and the energy stress induced in skeletal muscle during exercise, AMPK phosphorylation and downstream signaling was assessed in skeletal muscle at rest and following a maximal running test at a 5° incline to determine if alterations in AMPK α and β2 subunit content and/or downstream signaling contributed to reduced maximal running speed and altered substrate utilization in DKI mice (Figure 7A). Phosphorylation status of AMPK T172 relative to total AMPK α content in skeletal muscle was similar between genotypes at rest. While relative AMPK T172 phosphorylation was significantly higher in DKI mice compared to WT following a maximal running test, relative T172 phosphorylation was not significantly increased following exercise relative to the respective resting condition for either genotype (Figure 7B). Furthermore, exercise did not result in any significant differences in relative phosphorylation of AMPK β1 S182 in either DKI or WT mice (Figure 7C). There were also no differences in the relative phosphorylation status of AMPK’s downstream substrates between genotypes or between the resting versus maximally exercised conditions including phosphorylation of acetyl-CoA carboxylase (ACC) S79 (Figure 7D) and TBC1 domain family member 1 (TBC1D1) S660 (Figure 7E), an AMPK substrate involved in contraction-stimulated glucose uptake (Treebak et al., 2014; Stockli et al., 2015). However, total AMPK α (Figure 7F) and AMPK β2 (Figure 7G) protein content skeletal muscle from resting DKI mice, was reduced by 38 and 45%, respectively, compared to WT.

## Discussion

The primary findings of this study are that the DKI mutation utilized to chronically disrupt whole-body AMPK-glycogen binding reduced maximal running speed and resulted in increased depletion of glycogen levels in skeletal muscle following a maximal running test and was associated with reduced skeletal muscle AMPK α and β2 subunit content. Furthermore, DKI mice displayed increased rates of CHO oxidation during submaximal running, without any accompanying changes in substrate availability, impairments in skeletal muscle relative AMPK phosphorylation and downstream signaling at rest and following a maximal running test, or content of GLUT4, CPT1b and mitochondrial proteins. Collectively, these data suggest that the DKI mutation and the associated reductions in skeletal muscle AMPK α and β2 and/or glycogen content following exercise led to reduced maximal running speed in DKI mice.

It is well established that tissue AMPK α and β2 content, particularly in skeletal muscle, is associated with maximal exercise capacity. Loss of AMPK α in mouse muscle (Lantier et al., 2014; Fentz et al., 2015; Hingst et al., 2020), but not liver (Hughey et al., 2017), resulted in reduced maximal running speed. Furthermore, the β subunit, responsible for scaffolding the AMPK heterotrimer, has also been phenotypically linked with exercise capacity in mice. Muscle-specific β1β2 KO (O'Neill et al., 2011) and whole-body β2 KO (Steinberg et al., 2010; Dasgupta et al., 2012) mouse models exhibited reduced maximal running speed. Similarly, AMPK KI mice with disrupted glycogen binding capacity in the β2 subunit isoform, which is predominantly expressed in skeletal muscle, demonstrated reduced maximal running speed. In contrast, when glycogen binding capacity was disrupted in the β1 subunit isoform, the isoform predominantly expressed in liver, maximal running speed in mice was not impaired (Hoffman et al., 2020). Based on these skeletal muscle- and β2 isoform-specific roles of AMPK in exercise capacity and the positive correlation between change in muscle glycogen content and maximal running speed observed in WT mice in the present study, we focused our analyses of AMPK content and exercise signaling to skeletal muscle. Together with findings from previous studies, our current results suggest that the DKI mutation to disrupt AMPK-glycogen binding, specifically KI mutation of the β2 subunit isoform, reduces maximal running speed but not time to exhaustion at a submaximal speed.

Within skeletal muscle, AMPK phosphorylates a number of downstream targets that can influence tissue substrate utilization, including ACC and TBC1D1 which promote fat oxidation and contraction-stimulated glucose uptake, respectively (Fullerton et al., 2013; O'Neill 2013; Stockli et al., 2015). Previous studies have found differing exercise-mediated effects on relative AMPK phosphorylation and downstream signaling in WT mice dependent on the exercise protocol employed and/or muscle fiber type studied. For example, submaximal, steady state exercise resulted in significant increases in relative phosphorylation of AMPK T172 in glycolytic quadriceps muscle, which was associated with increases in absolute phosphorylation of both ACC S212 and TBC1D1 S231 (Hingst et al., 2020). However, no significant increases in relative phosphorylation of AMPK T172 or ACC S79 were observed in mixed gastrocnemius muscle in response to an incremental maximal running test similar to the protocol utilized in the current study (Hoffman et al., 2020). As a result, it is perhaps not surprising that a maximal running test at a 5° incline in the current investigation did not increase the relative phosphorylation of AMPK, ACC or TBC1D1 in WT mixed gastrocnemius muscle in the current investigation. Furthermore, the increased phosphorylation of AMPK T172 relative to total protein in DKI versus WT mice observed following a maximal running test did not result in any detectable changes in downstream phosphorylation of substrates ACC and TBC1D1 between genotypes, suggesting that substrate utilization patterns were not likely driven by changes in skeletal muscle AMPK phosphorylation and downstream signaling in response to exercise. Longer bouts of high intensity running (e.g., at higher running speed and/or treadmill incline) may be required to detect increases in relative phosphorylation in skeletal muscle of WT and DKI mice and/or elucidate potential impairments in relative AMPK phosphorylation and downstream signaling to other AMPK substrates in DKI skeletal muscle. We have previously shown that DKI mice exhibit increased adiposity concomitant with increased rates of whole-body CHO oxidation in the fed and resting state (Janzen et al., 2021), as well as during submaximal exercise in the present study. Therefore, it is possible that increased rates of CHO oxidation in DKI mice were not due to an acute shift in substrate oxidation patterns in response to exercise, but rather a continuation of chronically increased reliance on CHO substrate. Further study of adipose tissue and fatty acid production/utilization is warranted to determine if impairments in fat metabolism are contributing to increased rates of CHO oxidation in DKI mice. Alternatively, as the present study focused on mixed gastrocnemius muscle, it is possible that exercise-induced AMPK phosphorylation and signaling to downstream substrates could be differentially affected in other muscle fiber types in DKI mice and/or DKI mice may exhibit a shift in myosin heavy chain expression as a result of the DKI mutation leading to the observed changes in whole-body substrate utilization patterns. Future studies should evaluate if muscle fiber composition, GLUT4 translocation, or the regulation of other proteins involved in the glycolytic and β-oxidation pathways in various skeletal muscle groups (e.g., the pyruvate dehydrogenase complex and sarcolemmal fatty acid transporters) are affected as a consequence of disrupting whole-body AMPK-glycogen binding.

Findings regarding the role of different AMPK subunits and isoforms in the regulation of substrate utilization patterns in response to exercise are equivocal. In contrast to our findings of increased rates of CHO oxidation in DKI mice during submaximal running, recent work using an inducible skeletal muscle-specific AMPK α KO model has shown a reduction in maximal exercise capacity and increased ATP depletion, but no changes in rates of whole-body CHO or fat oxidation (Hingst et al., 2020), suggesting that skeletal muscle AMPK may not directly affect whole-body substrate utilization patterns. Additionally, mice with chronic KO of AMPK γ3, which is predominately expressed in glycolytic skeletal muscle, have unimpaired glycogen utilization following swimming exercise and normal contraction-stimulated glucose uptake in glycolytic extensor digitorum longus muscle (Barnes et al., 2004). Chronic muscle-specific AMPK β1β2 KO mice displayed increased reliance on fat utilization (O'Neill et al., 2011), while chronic muscle-specific AMPK α KO mice demonstrated an increased RER and impaired FA oxidation (Fentz et al., 2015) during submaximal treadmill running. DKI mice displayed smaller reductions in muscle AMPK α content than those observed in inducible skeletal muscle-specific AMPK α KO (Hingst et al., 2020), chronic whole-body AMPK γ3 KO mice (Barnes et al., 2004), muscle-specific AMPK β1β2 KO (O'Neill et al., 2011) and muscle-specific AMPK α KO mice (Fentz et al., 2015). Therefore, the relative AMPK α content remaining in DKI mouse skeletal muscle was likely sufficient to sustain rates of whole-body fat oxidation during exercise. The contrary findings in whole-body substrate utilization observed in DKI mice during energy stress compared to other whole-body, muscle-specific and/or inducible AMPK KO models may also be due either to chronically reduced AMPK α and β2 content in DKI mice and/or reduced AMPK levels observed in additional metabolic tissues, including liver and/or adipose tissue (Janzen et al., 2021). These differences in substrate oxidation patterns during exercise across these various transgenic AMPK mouse models highlight the limitations of current KO and KI models to study respective protein function and underscore the value of developing new inducible and tissue-specific models to elucidate the roles of AMPK and its glycogen binding capacity *in vivo*.

Both WT and DKI mice displayed similar increases in blood glucose concentration following a maximal running test at a 5° incline versus rest, suggesting that hepatic glucose output exceeds skeletal muscle glucose uptake (Camacho et al., 2005) and that DKI mice maintained hepatic glucose output and skeletal muscle glucose uptake during maximal exercise. Given the relatively large stores of liver glycogen, rodents rely predominantly on hepatic glycogenolysis to maintain blood glucose levels in settings of energy stress, such as following fasting or exercise, while the contribution of skeletal muscle glycogen to total energy expenditure becomes higher with increasing exercise intensity and/or duration (Baldwin et al., 1973; Pederson et al., 2005b). Therefore, given that DKI mice had a similar change in liver glycogen following a maximal running test - despite a shorter running time compared to WT mice (i.e., ∼19 min for DKI versus ∼29 min for WT mice)–it was likely that increased liver glycogenolysis was the primary contributor to the increased rates of CHO oxidation in DKI mice. However, there was no correlation observed between change in liver glycogen content and maximal running speed in either WT or DKI mice. The ∼60% reduction in liver glycogen observed in both genotypes following a maximal running test was therefore likely not limiting for achieving maximal running speed, suggesting that both WT and DKI mice possessed liver glycogen stores in excess of what could be utilized during an incremental maximal running test. Previous research in AMPK α KO mice has demonstrated impaired liver glycogenolysis, resulting in reduced hepatic glucose output during exercise without altering maximal running speed (Hughey et al., 2017). While findings from this previous study suggests that loss of liver AMPK results in impaired glycogenolysis and an associated reduction in hepatic glucose output, our current findings in DKI mice suggest that the hepatic AMPK α content lost as a result of the DKI mutation (Janzen et al., 2021) may not be critical for maintaining liver glycogenolysis and glucose output, and/or the remaining AMPK α content in DKI mice was sufficient to maintain required levels of hepatic glycogenolysis during treadmill running.

Due to the larger total contribution of liver glycogen to total energy expenditure during exhaustive exercise, it has been speculated that skeletal muscle glycogen is not a major determinant of exercise capacity during exhaustive exercise in mice. Previous studies have shown that chronic glycogen synthase KO mice lacking skeletal muscle glycogen did not have impaired running endurance capacity (Pederson et al., 2005b), while elevated skeletal muscle glycogen content via transgenic overexpression of constitutively active glycogen synthase did not enhance running endurance (Pederson et al., 2005a). Furthermore, skeletal muscle glycogen may not play a major role in whole-body rates of CHO oxidation and glucose homeostasis during exercise in mice (Irimia et al., 2010). However, in the present study we found a significant positive correlation between change in skeletal muscle glycogen and maximal running speed in WT mice. In contrast, there was no significant correlation between maximal running speed and change in skeletal muscle glycogen in DKI mice, concomitant with reduced muscle AMPK α and β2 content. In the current study we used an incremental maximal treadmill running test at a 5° incline, rather than endurance-based protocols employed in previous studies (Pederson et al., 2005a; Pederson et al., 2005b), and the contrasting results between the current work and previous studies could be explained by differences in exercise intensity and/or duration resulting in different contributions of glycogen to total energy expenditure. Therefore, it is possible that employing exercise protocols similar to the above studies and/or higher intensities could further elicit genotypic differences. Collectively, our results in the present study indicate that the ability to use skeletal muscle glycogen may potentially play an important role in achieving higher maximal running speed in WT mice.

The absence of a significant correlation between estimated levels of skeletal muscle glycogenolysis and maximal running speed at a 5° incline suggests that regulation of skeletal muscle glycogenolysis may be disrupted in DKI mice, particularly in response to energy stress. This contention is supported by previous results using this mouse model, which demonstrated increased skeletal muscle glycogen utilization during fasting in DKI mice (Janzen et al., 2021). It is possible that glycogen bound to AMPK in skeletal muscle may be protected from glycogenolysis, reciprocal to the proposed hypothesis that glycogen binding anchors cellular AMPK and prevents its degradation (Hoffman et al., 2020). Therefore, it is possible that the reduction in maximal running speed in DKI mice may not be directly related to the observed increases in rates of whole-body CHO oxidation and increased liver and skeletal muscle glycogen utilization, but rather may be due to indiscriminate utilization of skeletal muscle glycogen as a result of reduced skeletal muscle AMPK α and β2 content and/or glycogen binding capacity. Alternatively, it is possible that skeletal muscle glycogen utilization during exercise may be increased in DKI mice due to increased activity or capacity of the glycolytic pathway and/or alterations in muscle fiber type composition. Together, our findings suggest an important role for AMPK in determining acute exercise responses and maximal running speed independent of changes in whole-body substrate utilization patterns, which is consistent with findings in skeletal muscle AMPK KO mouse models (O'Neill et al., 2011; Hingst et al., 2020).

In conclusion, the results from the present study demonstrate that the DKI mutation used to disrupt AMPK-glycogen binding impairs maximal running speed and alters patterns of substrate utilization during submaximal exercise. Additionally, reductions in tissue AMPK α and β2 content are associated with increased utilization of skeletal muscle glycogen, resulting in increased rates of whole-body CHO oxidation. While increased CHO oxidation is not likely to be solely responsible for the observed reduction in maximal running speed, the DKI mutation results in indiscriminate utilization of skeletal muscle glycogen in mice, likely contributing to premature exhaustion during maximal running tests. These findings further underscore that AMPK serves additional roles in regulating tissue glycogen dynamics and adds another dimension to the known roles of AMPK in regulating substrate utilizations patterns and exercise capacity.

## Material and Methods

### Animal Models

AMPK β1(W100A)/β2(W98A) DKI mice on a C57BL/6J background were generated using CRISPR/Cas9 gene targeting, as described previously (Janzen et al., 2021). Homozygous carriers of both β1 and β2 isoform KI mutations were used for breeding, and DKI and WT breeders were annually backcrossed to generate heterozygous mice and rederive the homozygous DKI line. Confirmatory genotyping was performed from tail samples by Transnetyx (Cordova, TN, United States).

All experiments were undertaken using male DKI mice and age matched WT controls. Male mice were used to maintain adequate female breeders and ensure sufficient age matched litters were available for analyses. Mice were group housed in a temperature (∼22°C) and humidity-controlled facility with a 12:12 h light and dark cycle and given *ad libitum* access to a standard chow diet (6% fat, 20% protein and 29% starch; Barastoc, Ridley Agriproducts, Pakenham, Victoria, Australia) and water. All experiments commenced between 0,800 and 0,900 h when mice were in the fed state, unless otherwise stated. All mouse procedures were reviewed and approved by the St. Vincent’s Hospital (Melbourne, Victoria, Australia) Animal Ethics Committee (025-15 and 011-19), conformed to all the requirements of the National Health and Medical Research Council of Australia (NHMRC), and were in accordance with the Australian code of practice for the care and use of animals for scientific purposes (8th Edition 2013).

### Maximal Running Speed and Time to Exhaustion

Maximal running speed and time to exhaustion were assessed on a Columbus Instruments Exer 3/6 rodent treadmill (Columbus Instruments, Columbus, OH, United States). Mice underwent 4 days of treadmill acclimatization, as described previously (Hoffman et al., 2020). On day 5, mice completed an incremental maximal exercise capacity test to determine maximal running speed. Mice ran for 2 min at 10 m min^−1^, after which the speed was increased by 1 m min^−1^ every 2 min until exhaustion, defined as the inability to compel the mouse to remain running despite continued manual prodding. After 2–3 days of recovery, mice either completed the exercise calorimetry experiments (described below) or underwent a time to exhaustion test whereby they ran at 70% of individual maximal speed. Mice with similar maximal running speeds (i.e., within 2 m min^−1^) were grouped and completed their time to exhaustion tests simultaneously. These exercise tests were completed at a 0° treadmill incline (Table 1) and mice were returned to their home cages once testing was completed.

**TABLE 1:** Specifics of the four exercise protocols used and corresponding measures.

Exercise Protocol	Speed	Incline	Duration	Corresponding Measure and Figures
Maximal running speed	2 min at 10 m min^−1^ then speed increased 1 m min^−1^ every 2 min	0°	To exhaustion	- Maximal running speed (Figure 1A)
Time to exhaustion	70% of individual maximal running speed	0°	To exhaustion	- Time to exhaustion (Figure 1B)
Exercise calorimetry	60% of individual maximal running speed	0°	15 min (final 5 min used for calorimetry analysis)	- VO_2_, TEE, RER and rates of CHO and fat oxidation (Figure 2)
Maximal running test at a 5° incline	2 min at 10 m min^−1^ then speed increased 1 m min^−1^ every 2 min	5°	To exhaustion	- Blood glucose, blood lactate and serum NEFA (Figure 3)
				- Change in tissue glycogen (Figure 4)
				- Correlation between maximal running speed and change in tissue glycogen content (Figure 5)
				- Skeletal muscle AMPK α and β2 content and AMPK, ACC and TBC1D1 signaling (Figure 7)

A separate cohort of mice underwent acclimatization as described above but completed a maximal running test at a 5° incline (Table 1). Blood glucose and lactate were measured via tail tip bleeding the morning before and immediately following the maximal running test using an Accu-Check Performa glucometer (Roche Diagnostics GmbH, Mannheim, Germany) and Lactate Pro analyzer (Arkray, Kyoto, Japan). Mice were culled via cervical dislocation immediately following the maximal running test, and tissues were excised, immediately snap frozen in liquid N_2_, and stored at −80°C until subsequent biochemical analysis. Control rested mice did not undergo the maximal running test and remained in their home cage until being culled in the fed state at 0,900 h.

### Exercise Calorimetry

After completing the acclimatization and maximal running speed assessment at a 0° incline, a cohort of mice underwent an exercise bout to determine substrate utilization (Table 1). For exercise calorimetry experiments, mice completed 15 min running at 60% of their individual maximal speed at a 0° incline on a specialized rodent calorimetry treadmill (Omnitech Electronics, Columbus, OH, United States). Mean values for RER (VCO_2_·VO_2_^−1^) were determined. TEE and fat and CHO oxidation rates were calculated as previously described (Peronnet and Massicotte 1991).

### Serum Analysis

Blood samples were collected retro-orbitally immediately prior to and upon completion of a maximal running test at a 5° incline. Samples were left to clot for 30 min at room temperature in non-coated polypropylene tubes, then centrifuged at 10,000 *g* for 5 min at 4°C, and the serum supernatant was aliquoted and stored at −80°C. NEFA levels were measured using a NEFA-C kit (Wako Pure Chemical Industries, Osaka, Japan) according to manufacturer’s instructions.

### Tissue Glycogen Analysis

Liver and gastrocnemius muscle samples (∼25 mg) were chipped under liquid N_2_, freeze-dried overnight, powdered, and dissected free of visible blood and connective tissue. Aliquots of powdered tissue (∼2–4 mg) were then alkaline extracted and the supernatant was used for quantification of glycogen content using spectrophotometry, as described previously (Bergmeyer, 1974). Absorbance was measured at 340 nm using a SpectraMax Paradigm plate reader and SoftMax Pro microplate data acquisition software (Molecular Devices, San Jose, CA, United States).

### Tissue Protein Analyses

Gastrocnemius muscle samples were lysed in homogenization buffer containing 50 mM Tris-HCl (pH 7.5), 1 mM EDTA, 1 mM EGTA, 10% glycerol, 1% Triton-X, 50 mM sodium fluoride, 5 mM sodium pyrophosphate with cOmplete Protease Inhibitor Cocktail and PhosSTOP phosphatase inhibitor (Sigma-Aldrich, St. Louis, MO, United States). Samples were centrifuged at 16,000 *g* for 30 min at 4°C and protein content of the supernatant was determined using bicinchoninic acid (BCA) analysis (Pierce, Rockform, IL, United States). Lysates (10 µg protein·well^−1^) were run on 4–15% or 4–20% precast stain-free gels (Bio-Rad, Hercules, CA, United States) and transferred to PVDF membranes (Merck Milipore, Burlington, MA, United States). Membranes were blocked with 7.5% BSA in Tris-buffered saline containing 0.1% Tween 20 (TBS-T) for 1 h at room temperature then incubated with primary antibodies (1:1,000) overnight with rocking at 4°C. After washing with TBS-T, membranes were incubated with secondary antibody for 1 h at room temperature. Proteins were detected via chemiluminescence using SuperSignal West Femto Maximum Sensitivity Substrate (Thermo Fisher Scientific, Waltham, MA, United States) and imaged using the ChemiDoc Imaging System (Bio-Rad). Band intensities of total proteins and phosphorylation sites were normalized to the total lane protein from the respective stain-free image using Image Lab software (version 6.1, Bio-Rad), as previously described (Tachtsis et al., 2020). Protein phosphorylation was normalized to total content of the respective protein.

Antibodies against total AMPK α (2532), phospho-AMPK T172 (2531), total AMPK β (4150), phospho-AMPK β1 S182 (4186), total ACC (3662), phospho-ACC S79 (11,818), GLUT4 (2213), total TBC1D1 (66,433), phospho-TBC1D1 S660 (6928) and horseradish peroxidase-conjugated anti-rabbit (7074) and anti-mouse (7076) IgG secondary antibodies were purchased from Cell Signaling Technology (Danvers, MA, United States). Citrate synthase (ab96600), CPT1b (ab134988) and OXPHOS rodent antibody cocktail (ab110413) were purchased from Abcam (Cambridge, United Kingdom).

### Statistical Analyses

Comparisons between genotypes were analyzed using unpaired two-tailed Student’s t testing (WT versus DKI) or two-way analysis of variance (ANOVA; WT versus DKI comparisons over time or condition) with Bonferroni post hoc testing applied where appropriate. Pearson’s product moment correlation was used to examine associations between maximal running speed at 5° and change in tissue glycogen content. All statistical analyses were completed using GraphPad Prism software (version 9, GraphPad Software, La Jolla, CA, United States). Significance was set at *p* < 0.05 and all data are presented as mean ± standard error of the mean (SEM).

## Data Availability

The raw data supporting the conclusions of this article will be made available by the authors, without undue reservation.

## Glossary

ACC: Acetyl-CoA carboxylase
AMPK: AMP-activated protein kinase
ANOVA: Analysis of variance
CBM: Carbohydrate binding module
CHO: Carbohydrate
CPT1b: Carnitine palmitoyltransferase Ib
DKI: Double knock-in
GLUT4: Glucose transporter type 4
KI: Knock-in
KO: Knockout
TBC1D1: TBC1 domain family member 1
NEFA: Non-esterified fatty acid
OXPHOS: Oxidative phosphorylation
RER: Respiratory exchange ratio
SEM: Standard error of the mean
TBST-T: Tris-buffered saline containing 0.1% Tween 20
TEE: Total energy expenditure
VCO_2_: Carbon dioxide production
VO_2_: Oxygen consumption
WT: Wild type
